# Planned Behavior in the United Kingdom and Ireland Online Medicine Purchasing Context: Mixed Methods Survey Study

**DOI:** 10.2196/55391

**Published:** 2025-02-21

**Authors:** Bernard D Naughton

**Affiliations:** 1 School of Pharmacy and Pharmaceutical Sciences Trinity College Dublin, University of Dublin Dublin Ireland; 2 Centre for Pharmaceutical Medicine Research School of Cancer and Pharmaceutical Science Kings College London London United Kingdom

**Keywords:** planned behavior, consumer behavior, perceived behavioral control, attitudes, online purchasing, medicine

## Abstract

**Background:**

Online medicine purchasing is a growing health care opportunity. However, there is a scarcity of available evidence through a behavioral lens, which addresses why consumers buy medicines online. Governments try to influence online medicine purchasing behavior using health campaigns. However, there are little data regarding specific online medicine purchasing behaviors to support these campaigns.

**Objective:**

The theory of planned behavior explains that perceived behavioral control (PBC), attitudes, and norms contribute to intentions, leading to behaviors. This study challenges these assumptions, by testing them in an online medicine purchasing context. We asked: What is the role of attitudes, norms, and PBC in an online medicine purchasing context.

**Methods:**

An anonymous online snowball convenience sample survey, including open and closed questions concerning online medicine purchasing, was implemented. The data were thematically analyzed until data saturation. The emerging themes were applied to each individual response, as part of a case-by-case narrative analysis.

**Results:**

Of the 190 consumers from the United Kingdom and Ireland who consented to participate in the study, 46 participants had purchased medicines online, 9 of which were illegal sales. Of the 113 participants who demonstrated an intention to purchase, 42 (37.2%) completed a purchase. There were many cases in which participants demonstrated an intention to buy medicines online, but this intention did not translate to a purchasing behavior (71/190, 37.4%). Reasons for consumers progressing from intention to behavior are suggested to be impacted by PBC and attitudes. Qualitative data identified access to medicine as a factor encouraging online medicine purchasing behaviors and a facilitator of behavior transition. Despite understanding the importance of why some medicines required a prescription, which is described as an example of legal and health norms, and despite suspicion and concern categorized as negative attitudes in this paper, some participants were still buying products illegally online. Risk reduction strategies were performed by 17 participants (17/190, 9%). These strategies facilitated a transition from intention to behavior.

**Conclusions:**

The study results indicate that a consumer’s intention to buy does not automatically translate to a purchasing behavior online; instead, a transition phase exists. Second, consumers followed different pathways to purchase and used risk reduction practices while transitioning from an intention to a behavior. Finally, owing to the covert nature of online medicine purchasing, norms do not appear to be as influential as PBC and attitudes in an online medicine purchasing setting. Understanding how a consumer transitions from an intention to a behavior could be useful for researchers, health care professionals, and policymakers involved in public health campaigns. We encourage future research to focus on different consumer behavior pathways or ideal types, rather than taking a blanket approach to public health campaigns.

## Introduction

### Background

Authenticity is an important consideration when buying any product online, especially medicines, as inappropriate access and poor-quality medicines can have catastrophic implications for human health [1], with some sales causing death [2,3] including several coroner report cases [4]. Furthermore, online medicine purchasing has increased in recent times due to increased convenience, access, and an improved consumer experience [5,6]. From 1999 to 2003 in the United States, there was an increase in online sales of prescription drugs from $160 million to $3.2 billion [7], and the act of purchasing medicine online is likely to increase in line with internet access. In the United Kingdom in 2020, 96% of households had internet access, up from 2017, when 90% of UK households had internet access, and 2006, when only 57% of households had internet access [8]. These increases in internet access have also influenced the rate at which goods and services are bought online, with 87% of adults having bought goods or services online in 2020, up from 53% in 2008 [8].

Despite the dangers and increasing rates of online medicine purchases, there has been a scarcity of research to examine online medicine purchasing behavior. This paper examined online medicine purchasing through a behavioral model. The theory of planned behavior literature describes that past behavior, attitudes, perceived behavioral control (PBC), and norms affect a consumer’s intention and that this intention leads to a behavior. We challenge this assumption in the online medicine purchasing context and propose that there are unexplored contributing factors that affect a consumer’s likelihood of moving from an intention to a behavior. We did not aim to consider specific process variables such as the role of internal and external drivers on the translation from intention to behavior. Instead, we argued the presence of a transition phase that exists between an intention and a behavior and the core factors that specifically influence this transition. Although we see the merit in more granular research, it was not within the scope of this paper to pursue this.

#### Theory of Planned Behavior

##### Overview

This article examined human behavior in an online medicine purchasing context. Through a theory of planned behavior lens, we asked: What does the relationship between intentions and behaviors look like when buying medicine online? The theory of planned behavior is a social cognitive model in psychology that concerns intentional behavior; it was constructed by Ajzen in 1985 [9] and 1991 [10] and was originally known as the theory of reasoned action [11-15]. The theory identifies 3 determinants of intentions: (1) the attitude toward the behavior, which can be either positive or negative; (2) subjective norms, which are a person’s perceptions of the social expectations to adopt a behavior; and (3) PBC, which is a person’s perception of how easy or hard it might be to perform a behavior [16]. Since the book published by Fishbein and Ajzen in 2010 [13], this theory has also been known as the reasoned action approach. Although this theory was constructed over 50 years ago, it is still used in contemporary times, with Hagger et al [17] applying the reasoned action approach to health behavior in 2018 and contributing “past behavior” to the framework [18].

##### Theory of Planned Behavior: Strengths and Limitations

The theory of planned behavior is the most influential and highly cited model for the research and prediction of human behavior and is therefore a suitable theory to apply in this study [19]. However, like most behavioral theories, it has its limitations and criticisms in the literature when used in qualitative research [19]. One of the main limitations, which we addressed in this study, is the intention-behavior gap, as several studies consider intentions to be a poor indicator of behaviors [18,20] even over short time frames. The theory also does not take into consideration emotions or irrationality. The absence of emotion is important, especially when dealing with the purchase of health products as one’s health or the health of a loved one can have significant emotional impacts [21]. The lack of a focus on irrational behavior is an area of the model on which this study touched [19].

#### Online Medicine Purchasing Context

The at-distance medicine sales context is different to in-person sales for a variety of reasons. In an at-distance sales environment, attitudes, which are affected by suspicion or distrust, are augmented due to diminished seller-consumer accountability and a lack of physical interaction between a consumer and a health care professional, a lack of trust between the consumer and seller, and a fear of online fraud, often due to reports of rogue online traders [22-25]. Furthermore, norms are silenced at the point of sale, as the purchase usually occurs in isolation and is removed from direct and indirect social influence due to consumers commonly purchasing and administering the medicine covertly. Finally, PBC is augmented in an online context, due to the ability to buy products that may be prohibited or restricted, resulting in some patients buying illegal medicines online that they would not otherwise have access to. This context causes an augmentation of PCB when compared to in-person purchasing. We wished to understand if the transition from intention to behavior is as straightforward as explained in the literature or if there is a further unexplored nuance when applied to the online medicine purchasing context [26].

The constructs of planned behavior are relevant to the purchase of counterfeit luxury goods [27-29]. There are also many studies in the medical sciences literature that investigate issues concerning online pharmacies [5,22,30-38]. Based upon our literature review, there is a scarcity of published research concerning online medicine purchasing behavior research examined through a theoretical lens. This lack of research may be due to difficulties accessing patients who are willing to admit to buying medicines online for fear their activity may be illegal.

As part of a preprint report of a qualitative study conducted in the United Kingdom in 2023, Almomani and colleagues [39] interviewed 20 participants who had bought prescription-only medicines (POM) from the internet after 2019. They indeed acknowledged the reluctancy of consumers to disclose such activity because of its illegal connotations. The same research group also explored consumers’ beliefs by reviewing news media articles [40]. During analysis, they applied the theory of planned behavior model and observed behavioral beliefs (risks and benefits of purchasing), social beliefs (social influencing factors [eg, health care professionals and influencers]), and control beliefs (facilitators of purchasing [eg, accessibility, medicine shortages]) as influencing the likelihood of people buying POMs online. Hall and Antonopoulos [41] also conducted some behavioral research in this context. They used a virtual ethnography approach that provided qualitative evidence of the link between falsified medicine and online sales through the advancement of the field of demand dynamics and provided qualitative data that support their propositions and the findings of this paper.

This work is insightful but could be built upon through a more in-depth use of theoretical lenses. The rise of internet purchasing, the effect of poor-quality medicines on health [2], and the risks associated with buying potent medicines on the web [34,37] make this research and context a challenging area of unique and increasing importance in online consumer behavior research, where qualitative data are difficult to attain. The aim of this study was to gain a broad understanding of online medicine purchasing behaviors through the lens of planned behavior. In doing so, we questioned the role of attitudes, norms, and PBC in an online medicine purchasing context and how they affect the intention-behavior gap.

## Methods

### Study Design

This study used a mixed methods survey study design that posed open and closed questions to 190 members of the public. Due to the illegal nature of some online medicine purchases and the challenges of obtaining data from this population, an online convenience snowball sample survey was implemented. This approach was chosen to encourage anonymous participation. This included broad open-ended and closed-ended questions regarding the purchase of medicines online using Google Forms (Multimedia Appendix 1). The theory was developed subsequent to data collection [42]. This mixed methods survey was designed according to the CHERRIES (Checklist for Reporting Results of Internet E-Surveys) checklist [43].

### Data Analysis

The study involved a qualitative relational analysis that used thematic evaluation and inductive reasoning to identify themes to help better understand the relationship between intentions and behaviors in the online medicine purchasing context. Quantitative analysis used percentages to depict descriptive quantitative data and was generated from closed answer questions. This quantitative data supported the qualitative inductive reasoning, which proposed a novel transition phase between intentions and behaviors and the factors affecting this transition. Based on the emerging themes, a case-by-case narrative analysis was performed to further understand how these themes affected individual consumer purchasing journeys. This involved analyzing each case to understand the role that each theme played in their transition from intention to behavior. Percentages were rounded to 1 decimal place.

### Sampling Methodology

The recruitment approach was intended for scale and breadth using an established methodology [44,45] involving a convenience snowballing sampling method. Convenience sampling has its advantages: It is a useful tool for exploratory research in areas where there is little or no existing literature or where resources are limited. Convenience sampling can also generate large amounts of data in a short space of time and can identify hard-to-reach populations, such as willing participants who have purchased medicines online [46]. The survey contained closed questions to record the cohort demographics and open questions concerning the purchase of online medicines to facilitate qualitative data analysis. The data were collected in spring 2018 prior to the COVID-19 pandemic, which is a study limitation and should be considered within the context of that era. The inclusion criteria in this study were English-speaking internet users as per the methodology in [45].

### Study Recruitment

This was an open survey disseminated via social media. Therefore, this study was specifically representative of social media users and may not be representative of the wider public. The survey took approximately 10 minutes to complete, and the data were stored in an online database. No personally identifiable information was gathered. An invite to this survey was posted internally by researchers’ academic affiliations and the lead researcher’s professional LinkedIn and X (previously known as Twitter) and personal Facebook accounts. Reposting was encouraged to promote a snowballing effect. The survey contained a review step that allowed participants to go back to amend their responses. Cookies and IP checks were not used in this study. Only completed surveys were analyzed. The survey was posted and reposted through social media outlets for 4 weeks by the lead researcher, his contacts, and their connections. Payments or incentives were not provided to participants.

### Study Pilot and Consent

An anonymous, online, mixed methods survey, with a randomized response recording, was developed by the lead researcher and piloted with members of the research team and professional colleagues of the lead researcher to improve the survey questions. This included a review to ensure the online survey operated as intended, the questions were clear, and the survey was free of typos or grammatical errors (Multimedia Appendix 1). As part of the consenting process, the participants read study information and ticked a box consenting to being involved in the research. (Multimedia Appendix 1).

### Product and Sale Type

A free-text option in the survey allowed participants to identify the medicine or medicine type that they purchased online. These medicines were separated into the 3 most common UK legal classes: POM, pharmacy-only (P) medicines, and general sales list medicines. Some medicine classes differ based on the strength, quantity, formulation, and country in which they are purchased. Therefore, the medicines mentioned in this study were listed according to the most frequent class for each product in the United Kingdom. The medicines type, whether clinical questions were asked at the time of purchase, and whether a prescription was required were recorded to understand if the medicine was purchased legally or illegally. For the purposes of this study, an illegal sale was classified as one in which consumers were not asked for a prescription or asked medical questions when buying products that were considered as POM within the purchasing country.

A P medicine is one that does not require a prescription but can only be sold in a pharmacy. A POM medicine is one that requires a prescription and can only be supplied in a pharmacy. In cases where medicine class was uncertain (such as when the drug was listed as “Hair loss,” which could be a P medicine (minoxidil) or POM medicine (finasteride), the drug was classified as P.

### Ethical Considerations and Approval

This research was classified according to the National Institute for Health and Care Research guidelines. It was reviewed and received ethical approval from Keele University (reference number ERP2369). Participants provided informed consent to participate. Study data were anonymous. Participants did not receive compensation for participation.

## Results

This study involved a survey that generated qualitative and quantitative results that informed the contributions of this paper. The key themes were subjective norms, attitudes, and PBC.

### Demographics

This study attracted 227 respondents, of which 190 were living in the United Kingdom or Ireland and consented to the study (Table 1). The largest group (78/190, 41.1%) of participants was aged between 30 years and 39 years. There were more female respondents than male respondents (Table 1). The majority (135/190, 71.1%) of respondents lived in the United Kingdom, and 28.9% (55/190) lived in Ireland (Table 1). The study also included respondents with a variety of employment statuses and education levels. The majority (170/190, 89.5%) of respondents were employed full time or part time. Full-time students (13/190, 6.8%) and the unemployed (7/190, 3.7%) were also represented (Table 1). About one-third of participants (55/190, 28.9%) had health insurance, 64.7% (123/190) of participants lived in a country that provided free health care, and 6.3% (12/190) of participants did not have health insurance and did not live in a country that provided free health care.

**Table 1:** Demographic characteristics of the study participants (N=190).

Characteristics			Results, n (%)
**Gender**			
	Male	67 (35.3)	
	Female	123 (64.7)	
**Age (years)**			
	19-29	64 (33.7)	
	30-39	78 (41.1)	
	40-49	29 (15.3)	
	50-59	14 (7.4)	
	60-69	4 (2.1)	
	70-79	1 (0.5)	
**Country**			
	United Kingdom	135 (71.1)	
	Ireland	55 (28.9)	
**Employment status**			
	Full time	144 (75.8)	
	Part time	26 (13.7)	
	Full-time student	13 (6.8)	
	Unemployed	7 (3.7)	
**Education level**			
	Primary school	1 (0.5)	
	Secondary school	24 (12.6)	
	Undergraduate	85 (44.7)	
	Postgraduate	66 (34.7)	
	PhD	14 (7.4)	
**Health insurance status**			
	Free national health care	123 (64.7)	
	Health insurance	55 (28.9)	
	Pay for health care when required	12 (6.3)	

### Medicines Purchased

Of the 190 study participants, 46 had purchased medicines online. These medicines included a wide variety such as antibiotics, antimalarials, controlled drugs such as diazepam, and cosmetic medicines such as minoxidil for hair loss and Orlistat for weight loss (Multimedia Appendix 2). As all study participants were from the United Kingdom and Ireland, these medicines were categorized according to UK medicine classifications.

### Health and Legal Norms

The participants in this study were a largely well-educated population from the United Kingdom and Ireland (Table 1) who understood why prescriptions were needed. They could identify doctors, pharmacists, dentists, opticians, and nurses or a mixture of all 5 as the main professionals legally permitted to supply a prescription or drugs. The majority (189/190, 99.5%) of respondents knew that all medicines did not require a prescription and were aware of reasons why some medicines required a prescription and others did not. Respondents, which are numbered (#), gave many legitimate reasons why prescriptions were needed, for example:

Monitor drug usage,...ensure correct medications are given.

#159

To legally obtain drugs.

#152

To prevent risk to patients.

#141

To allow you to legally obtain medicines.

#79

Although the norm within the cohort was an understanding that prescriptions were required for the safe supply of medicine, of the 46 respondents who bought medicines online, 18 of those respondents (39%) purchased POMs (according to the UK classification). Of those 18 participants, 17 either had health insurance or free health care provided by the state. Of the 18 participants who bought POM online, 78% (17/18) were not asked for a prescription when purchasing these POM online, and 50% (9/18) were not asked for a prescription or asked medical questions when buying POM, making these 9 sales likely to be illegal. In summary, an interesting contradiction existed. Despite an understanding of the safety reasons for why prescriptions were needed, some participants still completed an illegal purchase of a medicine online without a prescription.

### Attitudes: Cost, Convenience, and Access

Participants were asked whether they would consider buying medicines online, and 59% (112/190) said that they would. In another question, participants were asked “On a scale of 1 to 10 (with 1 being unlikely and 10 being highly likely), how likely are you to buy a medicine online?” The total cohort gave an average mean response of 4.2, and those who had previously bought medicines online described a high likelihood, a mean average of 7.6, of buying medicines in the future.

There were several reasons in this study why consumers purchased their medicines online. Participants selected from a predetermined list of reasons for positive or negative attitudes that motivated consumers to buy medicine online, or they could free-type their response. The 2 reasons that contributed to positive attitudes toward online medicine purchasing behavior were convenience (18/46, 39%) and cost (22/46, 48%). A free-text space in this question resulted in 17 other responses. These responses largely focused on medicine access issues or due to their general practitioner (GP) not being willing to supply the medicine:

The specific medication that is most useful for my condition is not available in my country.

#162

Dr will only prescribe two weeks of treatment, despite chronic condition.

#66

The point relating to a GP’s unwillingness to supply (diazepam) was mentioned in this study a second time, when the same participant stated:

I was desperate as local GP will only prescribe very short-term treatment in low doses.

#66

Furthermore, despite buying and taking medicines bought online, 26% (12/46) of participants had negative attitudes toward online medicine purchasing. They explained that they were concerned that the medicine they purchased could be fake, yet these participants progressed to purchase.

### PBC: Risk Reduction Measures

The participants demonstrated that they had the autonomy and capacity to mitigate the risks associated with online medicine purchasing. In Table 2, we see a variety of practices used by our study participants to do so.

**Table 2:** Risk reduction practices used by this study population to support the online purchase of medicines.

Risk reduction practice	Example quotes (participant number)
No checks	“None, just had bought from it (online site) many times before but not medicines” (#34) “The product is manufactured from the same supplier that a prescription could have given me. They are cheaper, and a prescription is not required in this case. So, the safety of the website is not a factor to me.” (#137) “I assumed it was safe.” (#59) “None, because the product works (this is bad I know)” (#24) “Word of mouth reviews” (#4)
Recommendations and reviews	“By looking for recommendations on online forums” (#74) “Asked friends where they purchased from” (#3)
Using a well-known or reputable website or company	“It was the website of a pharmacy I used to shop when I lived there.” (#140) “Bought through an online pharmacy that is linked to a high street pharmacy. They asked a variety of health-related questions.” (#94) “Bought from Amazon, reliable source” (#183)
Other risk-mitigating methods	“Google search or company registration number check” (#158) “I conducted extensive research to ensure that the website was genuine, authentic, regulated and safe.” (#162) “Research the company I was buying from. Check their address on Google earth, recognise the name of the company, ensure site was safe (Https) and not a redirect” (#144) “Checked their registration from link to gov (government) website” (#37) “Register with regulator GMP (Good Manufacturing Practice) approved” (#175)

Practices to support a move from an intention to a purchasing behavior are categorized in this study as a form of PBC. From the cohort of 46 participants who purchased medicines online, there were a variety of methods used by respondents to support the purchasing behavior. These risk reduction practices are categorized as themes, represented as quotes, and summarized in Table 2. These practices include reading reviews and using a well-known or trusted website among other methods.

### Case-by-Case Narrative Analysis

As part of our case-by-case narrative analysis, which focused on the transition between intentions and behavior, we identified behavioral pathways or, as we call them, behavioral ideal types. In Table 3, we see that all participants in our study could be categorized into 6 patterns of behaviors or ideal types, which all involved an intention, an attitude (positive or negative), the presence or absence of risk reduction practices, and the presence or absence of the completed behavior. Although norms play an important role in a consumer’s intention to buy medicine online, they do not appear, in our data, to influence the transition from an intention to a purchasing behavior. This is possibly due to a lack of social influence at the point of purchase, as online medicine purchasing does not require social interaction with a pharmacist or other customers.

**Table 3:** The 6 ideal types identified in this study that describe the transition between intentions and behavior, mediated by attitudes and perceived behavioral control based on behavioral pathways revealed as part of the case-by-case narrative analysis (N=190).

Consumer ideal type	Intention	Attitude	Perceived behavioral control	Behavior	Participants, n (%)
1	Present	Unassessed	Unassessed	Absent	71 (37.4)
2	Present	Absence of a negative attitude	No risk reduction practices	Present	20 (10.5)
3	Present	Negative attitude	No risk reduction practices	Present	5 (2.6)
4	Present	Negative attitude	Risk reduction practices	Present	4 (2.1)
5	Present	Absence of a negative attitude	Risk reduction practices	Present	13 (6.8)
6	Absent	Unassessed	Unassessed	Absent	77 (40.5)

The case-by-case narrative analysis involved a review of each complete individual survey response to identify purchasing pathways from intentions to a purchasing behavior. On some consumers’ pathways from intention to behavior, they encountered a negative attitude postintention, and in some ideal types, they used practices to reduce risk. Consumers commonly had either no intention to purchase and did not (77/190, 40.5%; ideal type 6) or had intentions to purchase and did not (71/190, 37.4%; ideal type 1). These individuals were not asked questions regarding attitudes or PBC as they had no experience with online medicine purchasing. There was also evidence that participants with intentions to purchase had no negative attitudes toward the purchasing behavior; therefore, they did not need to implement a risk reduction practice and completed the purchase (20/190, 10.5%; ideal type 2). There were also participants (ideal type 3), who had an intention to purchase had a negative attitude toward the behavior, and, without reducing the risk, completed the purchase (5/190, 2.6%). There were also 4 participants (4/190, 2.1%) who had an intention to purchase, experienced a negative attitude, and used a risk reduction practice before completing the purchasing behavior (ideal type 4). Ideal type 5 (13/190, 6.8%) included individuals who had an intention to purchase, appeared to demonstrate no negative attitudes, implemented a risk reduction practice in any case, and completed the purchase. Ideal type 3 is an example of irrational behavior, as these study participants had an intention to purchase, then had negative attitudes toward the behavior, and, without taking any steps to manage that attitude, they still purchased. When asked “What measures, if any, did or do you take, to make sure the website that you are buying from is or was safe?”, one participant replied “None because the product works (this is bad I know)’ which typifies irrational behavior” (participant #24).

## Discussion

### Themes

**Figure 1:**
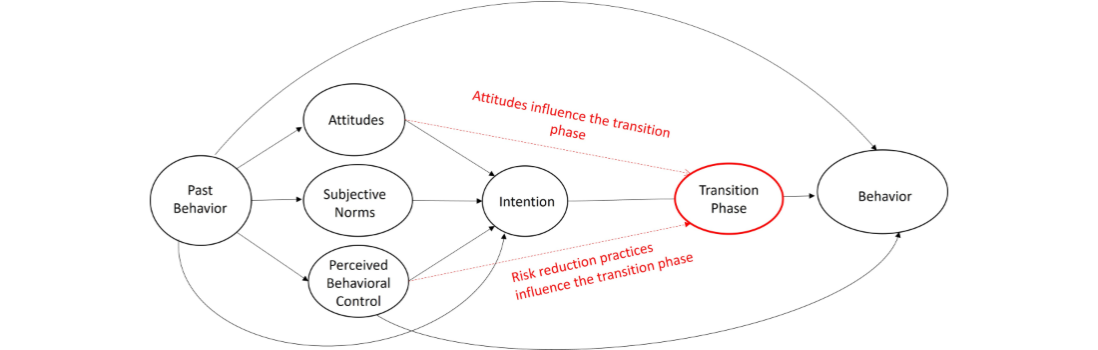
An adapted theory of planned behavior model with red lines representing this study’s proposed contributions, showing attitudes and perceived behavioral control as mediators of the transition from intention to behavior.

This study aimed to gain a broad understanding of online medicine purchasing behaviors through the theory of planned behavior lens with a focus on how intentions lead to behaviors and the intention-behavior gap. The dominant themes running through this paper concerned (1) health and legal norms; (2) attitudes toward online purchasing (positive: convenience and cost; negative: concern or suspicion); and (3) PBC, which presented as practices to minimize risk. The study also proposes that a transition stage (Figure 1) exists between intentions and behavior, which appears, in our data, to be influenced by attitudes and risk reduction practices but less so by norms.

### Theoretical Contribution

This paper identified qualitative and quantitative patterns within our data that support the use of the theory of planned behavior in this context [9,10] and described the influence of attitude, PBC, and norms on intentions and behavior. However, within this context, we suggest that the relationship between intention and behavior contains more nuance than is covered by Ajzen [9,10] and others [13,17]. We therefore adapted the model by Hagger et al [17] (Figure 1) to include our propositions, which are highlighted in red. Other studies support our proposal that intentions do not automatically translate to behaviors [26]. However, this paper reasons that a transition stage exists, where positive attitudes toward purchasing, such as convenience and cost, promote a transition from intention to behavior. Negative attitudes, which can emerge after the initial intention due to concern or suspicion, can inhibit a transition from intention to behavior [47], whereas PBC in the form of using risk reduction practices, such as reading reviews to mitigate negative attitudes, plays an important role in an actor’s successful psychological transition from an intention to a purchasing behavior. These propositions are supported in part by the work of Sniehotta [26] and aim to help address the intention to behavior gap in the literature (Figure 1).

This proposed transition phase is interesting and suggests that intentions can change before completing a purchase. If this transition stage exists, then there is an opportunity to influence consumers between the intention and behavior stages, which is an opportunity for public health campaigners or health care professionals to encourage or discourage practices that influence the transition phase.

### Norms: Health and Legal

There are several subjective norms that may affect online medicine purchasing behaviors. Norms differ in an online context because consumers have access to products that they would not normally have access to, due to availability issues or laws that prevent their purchase without a legal and valid prescription. In our study, the 2 most prominent norms that emerged included legal and health norms. There was no evidence from this study context that social norms negatively affected a participant’s transition from intention to behavior. This may be due to the standard medical treatment setting, where consumers are influenced by health care professionals to conform to norms during diagnosis and medicine supply, or by fellow consumers within stores. However, this is not the case online. Remote purchasing of medicine online is a step removed from the influences in a traditional purchasing environment, whereby the patient is without pressure to conform to social norms as they do not see the clinician or pharmacist face to face and are free to request whatever medicine they wish, in whatever quantity they desire. The effects of being refused a request online are unlikely to be as impactful as being refused in person by a health care professional who may know you and your family or have full access to your personal details and records. Therefore, it is understandable that the consumers in this study that transitioned from an intention to a behavior did not raise any negative normative influences throughout the transition.

If the medicine received from an online pharmacy is legitimate, the risks relate to addiction, inadequate medical supervision, and poor condition management. If the medicine is poor quality or falsified, there are additional risks such as side effects, overdose, and death. Buying medicine online is not a problem if the website is legitimate. Although several consumers answered yes to “At any stage were you ever concerned that the product(s) you were buying may have been fake, falsified, substandard or of poor quality?”, 7 of these (#3, #4, #24, #66, #74, #168, #182) still progressed to purchasing a product. From this evidence, we propose that the usual societal norms and legal medicine access norms in the online context do not appear to inhibit the transition from intention to behavior.

### Attitudes: Cost, Convenience, and Access

We present, in this paper, that attitudes toward purchasing behavior were influenced by cost, convenience, and access, which is also described in other studies [22].

Hall and Antonopoulos [41] described cases of patients purchasing medicines online due to inconvenience or because they were struggling to see a GP, when it was possible to see the GP:

After trying to get a doctor’s appointment for several weeks with no luck, I gave up and decided to go online to buy the drug I wanted.

The idea that consumers were taking control of the situation themselves due to cost and convenience was also covered in another study by Almomani et al [22]:

There is no need to make an appointment with your GP. Well, it takes a couple of weeks to get an appointment in our surgery.

2QL

Medicine access was a qualitative theme that commonly emerged from the survey data, which also correlates with a study by Cicero and Ellis [32] and is argued to impact attitudes. The 2 reasons for buying tramadol online in the empirical study by Cicero and Ellis were (1) difficulty finding a doctor to prescribe the opioid medicine and (2) doctors who would not prescribe enough. Both points are access-related issues that affect PBC, appear to be augmented in the context of online addictive drug purchasing, and increase intentions to purchase. In the case of addictive medicines, access appears to be a key driver of intentions. Another example from this study was related to diazepam, and in that case, the participant who bought the diazepam online also explained that they were motivated to do so because their doctor refused to supply enough of the medicine.

In this case, the participant went on to buy this medicine online from a website that did not require an online consultation or a prescription. This is a case of a patient bypassing the GP to obtain medicines, which demonstrates autonomy. This is a common case that is also reflected in quotes gathered by Hall and Antonopoulos in their 2016 study [41].

The issue of limited access affects the participant’s attitude toward the behavior and may be a driver to regain PBC that encourages online purchasing behavior which appears to be common in this study and the wider literature.

### PBC and Risk Reduction Practices

We propose that PBC affects the psychological transition from intention to behavior. Consumers can assert their own control in a purchasing context to mitigate against risks that develop postintention and prebehavior, such as a consumer noticing something suspicious about an online pharmacy. Their PBC allows them to check the website URL against a list of approved websites or check other customer reviews to mitigate their negative attitudes toward that purchase [48-51].

Hall and Antonopoulos [41] identified the importance of finding a trusted illegal source.

In order to find a “Reputable” supplier, a consumer commonly relied on the use of risk reduction practices before purchase, which is a form of PBC. These practices included their own research, the company’s reputation, and online reviews (Table 2). We reason that postintention risk reduction practices influence consumer transitions from intention to behavior. This is supported by evidence from Filieri et al [48] and Jin et al [49], who discussed the role of product reviews in online decision-making. When consumers encounter convincing information to mitigate identified risks, such as online reviews, they tend to complete the transition from initial intention to behavior [48]. Of course, there will be exceptions to this transition scenario, whereby consumers’ intentional and logical behaviors are overtaken by impulsiveness behaviors [52], which is covered by consumer ideal type 3 in Table 3.

### Behavioral Pathways: Public Health Policy Implications

A consumer’s transition from intention to behavior, depicted as consumer ideal types in Table 3, acts as a lens through which to see consumer purchasing behavior patterns. This study has its limitations; however, these ideal types could be useful tools to help understand how different consumer groups transition from an intention to a behavior. This may assist with targeting individual psychological processes associated with norms, negative attitudes, PBC, and the facilitation of behavioral change through targeted public health campaigns. The current public health campaign policies concerning the purchase of falsified medicine online but do not appear to consider the different attitudes and PBC practices that affect individuals. Instead, public health campaigns either send an educational message about the risks of buying medicines online or try to incite fear into customers to deter them from the online purchase of medicine. Viewing consumers through different ideal types will provide public health campaign marketers with a new structured strategy to help them target specific behavior types. For example, consumers who demonstrate a negative attitude toward the online purchasing behavior but tend to continue from intention to behavior when adopting a risk reduction practice are unlikely to find a public health campaign that incites fear or suspicion as an effective deterrent. In those cases, these individuals would be best served by public health campaigns that describe safe and effective risk reduction practices such as explaining ways to verify the legitimacy of the website or the medicine they are purchasing.

### Retail Pharmacy Marketing Implications

For legitimate online pharmacies, it may be useful to create marketing approaches that develop upon the principles described in this paper. These approaches may include the reassurance of consumers with negative attitudes and sign-posting reliable risk reduction practices to help reassure their customers. Reassurance may include regular blogs or posts concerning the risks of buying from illegal pharmacies; risk reduction practices to support patients, which may include searching for privacy or security statements [25]; or methods to verify online pharmacies or medicines they receive from pharmacies. These practices could be monitored using consumer metrics to understand which strategies were most effective at converting intentions to behaviors [53]. This argument may also hold true for marketers in other industries, as they too could run campaigns to raise awareness of illegal websites and ways to verify the legitimacy of an online retailer or the products they sell.

### Limitations

This study is based on a relatively small sample of participants from the United Kingdom and Ireland and could benefit from being repeated as part of a large, multicenter, triangulated, mixed methods study that includes interviews, focus groups, and observations across different regions. In addition, the data were collected in 2018; therefore, the findings should be considered in light of that era. This study was also based on social media users, who may not represent a suitable cross-section of society. However, considering the sensitive nature of the subject, it can be difficult to recruit participants to such a study. This study is a first step to identifying the presence of certain factors facilitating or inhibiting a consumer’s transition from an intention to a behavior in an online medicine purchasing context.

In conclusion, intentions do not always directly translate to a behavior in an online medicine purchasing context, which is an important consideration for researchers in this area. Instead, we suggest that the transition is mediated by PBC and attitudes. In many cases, those buying medicines online were concerned that their medicine may be a fake but still proceeded to purchase, which is concerning from a public health perspective. In cases where a complete transition from intention to behavior did occur, it was often in the absence of a negative attitude or the presence of a negative attitude coupled with a practice to reduce risk. The arguments in this article support the presence of a transition phase when buying medicine online. It is hoped that this paper’s contribution will help public health campaigners and online pharmacy marketers to successfully target specific behavior types and support the use of consumer risk mitigation tools.

## Appendix

Multimedia Appendix 1 The questionnaire applied in this study.

Multimedia Appendix 2 Medicines purchased by respondents in this study.

## Abbreviations

CHERRIES: Checklist for Reporting Results of Internet E-Surveys
GP: general practitioner
P: pharmacy-only
PBC: perceived behavioral control
POM: prescription-only medicines

## Data Availability

Primary study data are available on request.
