# Supplementary material for: Planned Behavior in the United Kingdom and Ireland Online Medicine Purchasing Context: Mixed Methods Survey Study
Source: JMIR Form Res. 2025 Feb 21;9:e55391. doi: 10.2196/55391 (PMC11890141; doi:10.2196/55391)
Supplement: Multimedia Appendix 1 [file formative_v9i1e55391_app1.docx]

**Appendix A: The questionnaire applied in this study**

**The Safe Purchase of Medicines Online: An Anonymous General Public Health Survey**

Data suggests that many online pharmacies around the world are operating illegally. Many of these pharmacies sell fake or poor-quality medicines, many others may be failing to provide adequate patient information. This compromises patient safety. This study is being conducted by researchers at X University and the University of X.

You have been invited to take part in this anonymous survey because you are a member of the general public that uses the internet. It does not matter if you have or have not purchased medicines online, you can still contribute. We would like you to complete this survey to help us understand more about three key issues which relate to online medicine purchases.

1. What would motivate you to buy medicines online? 2. What do you know about obtaining medicines safely? 3. What is your opinion on mobile phone apps which verify the status of a medicine, delivering education about buying medicines online and also providing healthcare advice?

Our research team is eager to hear your perspectives on these topics. Please take your time to answer this survey honestly. Completing this survey should take less than 10 minutes and will help our team to understand more about key issues relating to online medicine purchases. Participation in this survey is optional.

This is an anonymous survey and will be used for academic purposes only. The outcomes of this research will be published in a peer-reviewed journal to ensure this learning is shared with fellow researchers. Your data will be stored according to X University data handling policy. A layperson summary will also be distributed through the same mediums used to recruit participants. If you have any concerns about this study, please feel free to contact the lead researcher at xxxxxx@xxx.ac.uk. They will respond to your questions or concerns within 3 working days.

**Consent**

I understand that my participation is voluntary. However, once this survey is completed it will not be possible to withdraw at any time because my responses will be stored anonymously. I understand that research data collected during the study may be looked at by designated individuals from X University or the University of X where it is relevant. By taking part in this study, I give permission for these individuals to access the data contained within my survey response. I understand that this project has been reviewed by, and received ethics clearance through, the X University Research Ethics Committee. I understand who has access to the data I provide and that this data will be stored according to X University data handling policy. I understand how this research will be written up and published. I understand how to raise a concern or make a complaint.

**1.** Considering the statements above do you consent to participate in this study? *Mark only one oval. Yes No

**The Extent of the Problem2.** What percentage of online pharmacies do you think operate illegally? * Mark only one oval. Less than 5% 6-10% 11-20% 21-30% 31-40% 41-50% 51-60% 61-70% 71-80% 81-90% 91-100% **3.** Studies show that up to 97% of online pharmacies are operating illegally, are you surprised by this figure? * Mark only one oval. Yes No

**Demographic Questions**This section is designed to gather information about the participants of this study. This will help us to learn about the general level of education around medicine purchase, the types of people who buy medicines online, and where online medicine purchasing is most common.

The Safe Purchase of Medicines Online: An Anonymous General Public Health Survey

**4.** What is your age? * Mark only one oval. 0 - 12 13 - 15 16 - 18 19 - 24 25 - 29 30 - 39 40 - 44 45 - 49 50 - 59 60 - 69 70 - 79 80 - 89 90 - 100 Over 100 **5.** What is your gender? * Mark only one oval. Male Female Other (please state below) Other: ……………………….**6.** What is your level of education? * Mark only one oval. I have a Primary School education I have a Secondary School education I have an Undergraduate Degree I have a Postgraduate Degree I have a PhD Other: ……………….**7.** What is your employment status? * Mark only one oval. Full-time employed Part-time employed Unemployed Part-time Student

Full-time Student**8.** How do you pay for your healthcare? * Mark only one oval. I have health insurance Healthcare is free in my country My country does not provide free healthcare and I do not have health insurance, I pay for my healthcare myself when needed **9.** What is your ethnic origin? * Mark only one oval. British White White - Irish Irish Traveller Gypsy or Traveller Other White background Black or Black British - Caribbean Black or Black British - African Other Black background Asian or Asian British - Indian Asian or Asian British - Pakistani Asian or Asian British - Bangladeshi Chinese Other Asian background Mixed - White and Black Caribbean Mixed - White and Black African Mixed - White and Asian Other mixed background Other ethnic background Not known Information refused **10.** What is your country of nationality? * Mark only one oval. (List of all countries for selection)

**11.** Where do you currently live? * Mark only one oval. (List of all countries for selection) **12.** If you are from the United Kingdom or the US please state the first two or three figures of your postcode or zip code below, this will allow us to compare different regions of the UK and US in terms of medicines purchased online. e.g. Liverpool might be L5, Oxford might be OX1 or 100 might be New York. *

**Knowledge Relating to the Safe Supply of Medicines**(Each question is followed in brackets by Norms, Attitudes or PBC to signal alignment between that question and a specific theme or themes)

This section aims to understand your knowledge on the subject of "Safe Medicine Supply" **13.** Do you know what a prescription is? * (Norms)Mark only one oval. Yes No Not sure **14.** Do you think you always need to see the doctor to get a prescription? * (Norms)

Mark only one oval. Yes No Not sure **15.** Who else can write legal prescriptions? * (Norms) **16**. Who do you think is legally permitted to supply you with medicines in your country? * (Norms)**17.** Could you explain what you think a prescription is used for? * (Norms & PBC)

**18.** Do you think that all medicines require a prescription? *.(Norms)

Mark only one. Yes No Not sure **19.** Did you know that some medicines can be bought from a supermarket, some can be bought from a pharmacist without a prescription and some must always be obtained with a prescription? * (Norms & PBC)Mark only one oval. Yes No **20.** Why do you think there are restrictions on the sale of medicines and why some require a prescription, and some do not? * (Attitudes & PBC)**21.** Would you consider buying a medicine online? (This includes any medicine e.g. creams, ointments, injections, tablets, capsules, liquids, suspensions, inhalers, eye drops, ear drops etc.) *(Attitudes) Mark only one oval. Yes No

**22.** On a scale of 1 to 10 (with 1 being unlikely and 10 being highly likely), how likely are you to buy a medicine online? *Mark only one oval. 1 2 3 4 5 6 7 8 910 **23.** Have you ever purchased medicines online? (This includes any medicine e.g. creams, inhalers, tablets, injections etc. which you requested and paid for online) *(Behaviour) Mark only one oval. Yes No (Skip to question 38). **Medicines Purchased Online** In this section, we would like to understand more about the behaviour associated with buying medicines online. Please think about a time or times that you bought medicine online. **24.** Do you normally buy medicines online for yourself, your partner, a friend or a relative? (Behaviour) * Mark only one oval. Myself

My partner A friend A relative I have never bought medicine online **25.** What medicines are you most likely to buy online? (select option or options that apply) (Attitudes) Tick all that apply. Those for cosmetic conditions e.g. hair loss, weight loss or erectile dysfunction

Those with a long-term condition e.g. diabetes, asthma, arthritis or blood pressure control.

Those with a short-term condition e.g. pain relief or antibiotics for an infection Other: **26.** What devise did, or do you use to buy the medicine online? *(Behaviour) Mark only one oval. A smartphone A tablet A laptop

A desktop computer Asked someone else to buy them for you online

**27.** Please, could you describe the medicine(s) or type of medicine(s) you purchased? *….(Behaviour)**28.** What were the reasons for you buying your medicine online? (please select the option or options which apply) *(PBC, Attitudes, Norms, depending on answer) Tick all that apply. Physically unable to get to a pharmacy Buying medicine online is cheaper I have to wait too long to get an appointment with my doctor Buying medicine online is more convenient I was embarrassed about my condition I do not have health care provided by the state or health insurance, and cannot afford medicine from the local pharmacy Other: ………… **29.** At any point in the process did you think the drug(s) you were buying might be fake, counterfeit or of poor quality? *(Norms & Attitudes) Mark only one oval. Yes No Maybe Other:

**30.** Were you asked for a prescription? * (PBC)Mark only one oval. Yes No I cannot remember Other: **31.** Were you asked medical questions about your condition? * (PBC) Mark only one oval. Yes No Other:

**32.** Did you, or the person that these medicines were bought for, take these medicines? * (Behaviour)Mark only one oval. Yes No After the last question in this section (skip to question 35). I am not sure After the last question in this section (skip to question 35). **33.** Did you or the person that these medicines were bought for think that the medicines bought online resulted in any side-effects which were different from usual? * (Behaviour)Mark only one oval.

Yes

No, After the last question in this section, skip to question 35.Not sure After the last question in this section, skip to question 35.

**34.** If you believe the medicine caused side effects, could you explain what these were? *…(Behaviour) **35.** At any stage were you ever concerned that the product(s) you were buying may have been fake, falsified, substandard or of poor quality? * (Attitudes) Mark only one oval. Yes No **36.** What measures, if any, did or do you take, to make sure the website that you are buying from is or was safe? * …….(PBC)

**37.** What measures, if any, do you take, to make sure the medicine(s) you received was safe. (PBC)
