# Supplementary material for: Planned Behavior in the United Kingdom and Ireland Online Medicine Purchasing Context: Mixed Methods Survey Study
Source: JMIR Form Res. 2025 Feb 21;9:e55391. doi: 10.2196/55391 (PMC11890141; doi:10.2196/55391)
Supplement: Multimedia Appendix 2 [file formative_v9i1e55391_app2.docx]

###### **Appendix B: Medicines purchased by respondents in this study.**

| **POM** | **P** | **P, or POM** | **GSL** | **Supplements** |
| --- | --- | --- | --- | --- |
| Antibiotics | Orlistat® | Acne Cream | Bio-Freeze® | Chondroitin |
| Antimalarials | Minoxidil | Antacid | Silcocks base | Glucosamine |
| Blood pressure medication | Fungal nail infection ointment | Emergency Contraceptives | First Aid Kit supplies | Natural Desiccated thyroid supplement |
| Contraceptives | Paracetamol based cough medicine | Nappy Rash cream | Paediatric nasal saltwater | Garlic Supplements |
| Valium® (Diazepam) | Antihistamines | Fluconazole | Deep heat® | Co-enzyme |
| Galfer® (Ferrous Fumarate) | Calpol® (Paracetamol suspension) | Hair Loss medications |  | Herbal Ointments |
| Inhalers |  | Sildenafil |  | Omega 3 |
| Modafinil |  | Steroid Cream |  |  |
| Ventolin ® (salbutamol) |  | Weight loss tablets |  |  |
| Retin-A® (Tretinoin) cream |  | Voltarol (Diclofenac) |  |  |
